# Prevascularization promotes endogenous cell-mediated angiogenesis by upregulating the expression of fibrinogen and connective tissue growth factor in tissue-engineered bone grafts

**DOI:** 10.1186/s13287-018-0925-y

**Published:** 2018-07-04

**Authors:** Pengzhen Cheng, Donglin Li, Yi Gao, Tianqing Cao, Huijie Jiang, Jimeng Wang, Junqin Li, Shuaishuai Zhang, Yue Song, Bin Liu, Chunmei Wang, Liu Yang, Guoxian Pei

**Affiliations:** 1 Institute of Orthopedic Surgery, Xijing Hospital, Fourth Military Medical University, Xi’an, 710032 People’s Republic of China; 2 Hospital 463 of People’s Liberation Army, Shenyang, 110042 People’s Republic of China; 3 Department of Orthopedics, The 251st Hospital of PLA, Zhangjiakou, 075000 China

**Keywords:** Prevascularization, Angiogenesis, Tissue-engineered bone grafts, Fibrinogen, Connective tissue growth factor

## Abstract

**Background:**

Vascularization is one of the most important processes in tissue-engineered bone graft (TEBG)-mediated regeneration of large segmental bone defects. We previously showed that prevascularization of TEBGs promoted capillary vessel formation within the defected site and accelerated new bone formation. However, the precise mechanisms and contribution of endogenous cells were not explored.

**Methods:**

We established a large defect (5 mm) model in the femur of EGFP^+^ transgenic rats and implanted a β-tricalcium phosphate (β-TCP) scaffold seeded with exogenous EGFP^−^ cells; the femoral vascular bundle was inserted into the scaffold before implantation in the prevascularized TEBG group. Histopathology and scanning electron microscopy were performed and connective tissue growth factor (CTGF) and fibrin expression, exogenous cell survival, endogenous cell migration and behavior, and collagen type I and III deposition were assessed at 1 and 4 weeks post implantation.

**Results:**

We found that the fibrinogen content can be increased at the early stage of vascular bundle transplantation, forming a fibrin reticulate structure and tubular connections between pores of β-TCP material, which provides a support for cell attachment and migration. Meanwhile, CTGF expression is increased, and more endogenous cells can be recruited and promote collagen synthesis and angiogenesis. By 4 weeks post implantation, the tubular connections transformed into von Willebrand factor-positive capillary-like structures with deposition of type III collagen, and accelerated angiogenesis of endogenous cells.

**Conclusions:**

These findings demonstrate that prevascularization promotes the recruitment of endogenous cells and collagen deposition by upregulating fibrinogen and CTGF, directly resulting in new blood vessel formation. In addition, this molecular mechanism can be used to establish fast-acting angiogenesis materials in future clinical applications.

**Electronic supplementary material:**

The online version of this article (10.1186/s13287-018-0925-y) contains supplementary material, which is available to authorized users.

## Background

Large segmental bone defects caused by severe trauma, tumor, and pathological fractures usually fail to heal naturally due to the limited self-repairing capabilities [1, 2]. Over the last two decades, regenerative medicine and bone tissue engineering techniques have offered promising alternative approaches for the treatment of large bone defects without side effects compared with traditional therapies [3–6].

Vascularization is one of the most important processes in TEBG-mediated regeneration of large segmental bone defects. Angiogenesis is a highly regulated event involving complex, dynamic interactions between vascular endothelial cells and extracellular matrix (ECM) proteins [7]. Early neovascularization after TEBG implantation in vivo is a major obstacle to overcome in achieving satisfactory healing [8, 9].

In our previous studies, TEBGs were prevascularized by inserting the femoral arteriovenous bundle and then implanted to treat large bone defects. At 4 and 8 weeks after implantation, significantly more new capillary vessels had formed inside prevascularized TEBGs than in control scaffolds [9–11]. However, the exact mechanisms for this positive effect remain unknown. Significant research has been conducted to explore the function, survival, and homing of exogenous seed cells [12–15]. However, endogenous cells play a more important role in the process of bone repair, yet there are few studies on the role and function of endogenous cells [16–18].

In this study, we elucidated the mechanisms by which transplantation of the femoral vascular bundle into the TEBG promotes angiogenesis and vascularization of the scaffold, specifically focusing on the participation of endogenous cells in bone defect repair. We established a large segmental femoral defect in EGFP^+^ transgenic rats to investigate the recruitment and behavior of endogenous cells, by tracking the migration and survival of endogenous EGFP^+^ cells within the scaffold using fluorescence microscopy; the scaffolds were seeded with wild-type cells isolated from EGFP^−^ rats.

The β-TCP scaffold has spherical micropores with controllable size, and there are pore connections between the micropores to ensure that the connectivity rate is over 99%. The pore size and porosity of the material were suitable for cell adhesion and tissue ingrowth. In addition, the scaffold has good biocompatibility and no carcinogenicity [19, 20].

We report that prevascularization led to the formation of a reticulated fibrin structure throughout the scaffold, which accelerated the recruitment of endogenous cells and type III collagen deposition by inducing high-level expression of connective tissue growth factor (CTGF) and, in turn, promoted the formation of new blood vessels by endogenous cells within the TEBG. This study provides a new basis for further clinical research to develop novel TEBG materials and approaches.

## Methods

### Animals and ethics statement

EGFP^+^ transgenic (Sprague–Dawley (SD)) rats were purchased from Xing Ming Biomedical Technology Co., Ltd (Shanghai, China). Wild-type (WT, EGFP^−^) SD rats were obtained from the experimental animal center of Fourth Military Medical University, Xi’an, China. The rats were housed in a specific pathogen-free (SPF) animal facility at the animal center of Fourth Military Medical University.

All animal procedures were performed in the authorized animal care facility and were approved by the “Committee for the Care and Use of Laboratory Animals” of Fourth Military Medical University. All methods were performed in accordance with the relevant guidelines and regulations of the People’s Republic of China.

### Preparation of TEBGs

Porous β-tricalcium phosphate (β-TCP) scaffolds (70% porosity, 4 mm × 5 mm) with a 1-mm deep side groove were used to carry the implanted vascular bundles (Bio-lu Biomaterials, China). The β-TCP scaffold has spherical micropores with controllable size (450 ± 50 μm), and there are pore connections (150 ± 50 μm) between the micropores to ensure that the connectivity rate is over 99% [19]. The mechanical strength is greater than 2 MPa with no cytotoxicity and rejection.

EGFP^−^ BMSCs were isolated from the femoral bone marrow of 2-week-old wild-type rats. The culture medium was changed after the first 24 h, and then every 48 h the medium was replaced to remove the dead and nonadherent cells. When the cell density reached approximately 80%, the adherent cells were trypsinized, harvested, and seeded into new dishes at 1.0 × 10^5^ cells. Third-passage BMSCs (after 3 weeks of culture) were seeded onto the scaffolds at a density of 5 × 10^6^ cells/ml and the cell–scaffold complexes (TEBGs) were incubated for 7 days before transplantation.

### Large segmental bone defect model in the rat femur

The EGFP^+^ SD rats (female, 12 weeks old, 220 ± 10 g) were randomly divided into two groups with eight rats in each group: the prevascularized TEBG group and the TEBG group. The animals were fasted overnight before surgery and anesthetized by intraperitoneal injection of 2% w/v pentobarbital sodium salt (45 mg/kg; Merck, Germany).

We made rat bone defect models as described previously [21]. Briefly, after being shaved and sterilized, the rat’s femoral arteriovenous bundle was exposed by creating a 2-cm incision inside the left hind limb. The arteriovenous bundle and saphenous nerve were then separated by microsurgical techniques, carefully dissociating out about 20–25 mm. Next, blunt dissection of the fascia and muscle was performed to expose the left femoral shaft, and a large bone defect (5 mm in length) was created in the femur and fixed using an internal fixation plate. In the prevascularized TEBG group, we embedded the arteriovenous bundle into the β-TCP groove to construct a prevascularized TEBG complex, which was implanted into the defect site together. As a control, the TEBG group was implanted with β-TCP material at the defect site without embedding the vascular bundle.

The wounds were stitched and disinfected with iodophor. After the operation, X-ray imaging was performed at 35 kV and 1.5 mA for 3 s using a Carestream DRX Ascend (Carestream Health, Canada) to check plate fixation and ensure successful establishment of the critical bone defect. After recovery, all experimental animals were intramuscularly injected with 400,000 U penicillin/day for 3 days.

In order to study the migration and distribution of endogenous cells and the survival of exogenous seed cells in the repair of bone defects, we used WT-BMSCs (EGFP^−^) as exogenous cells to generate a β-TCP complex for further implantation into EGFP^+^ rats (all cells are EGFP^+^). Cell sources in the TEBGs can be determined by tracing green fluorescent protein.

### Histological analysis

At 1 and 4 weeks after operation, the femurs were carefully removed after cardiac perfusion and fixation, and secondary fixed in 4% paraformaldehyde (PFA) solution for 3 days. The samples were decalcified in 10% ethylenediaminetetraacetic solution (EDTA; Sigma, USA) for 6 weeks, with gentle shaking at room temperature (until the samples were soft enough to be easily punctured by a needle).

The femoral specimens were dehydrated in a mixture of 10% gum arabic and 30% sucrose for 3 days at 4 °C. After that, specimens were embedded in an OTC compound (Sakura Seiki, Tokyo, Japan) and serially sectioned perpendicular to the long axis of the femur using a cryostat (Jung CM3000; Leica, Wetzlar, Germany). Each section (8 μm thick) was fixed on a glass slide and preserved at − 20 °C.

Some sections were rewarmed, stained with hematoxylin and eosin (H&E), and observed by light microscopy (BX53; Olympus, Japan).

### Scanning electron microscopy analysis

At 1 week post operation, the femurs were carefully removed and fixed in solution containing 4% paraformaldehyde (PFA) and 3% glutaraldehyde (1:1) for 3 days. Inspection of the transversal surface of the material center (Hitachi-S3400N, Japan) used acceleration voltage ≥ 15 kV and imaging distance 5–10 mm.

### Immunofluorescence analysis

A random selection of some sections from each group were rewarmed at 37 °C for 30 min, wetted with PBS for 3 min, then permeabilized with 0.2% Triton X-100 (Sigma, USA) for 10 min at room temperature, washed three times with PBS, and incubated in blocking agent (10% donkey serum; Solarbio) to block nonspecific binding. Sections were incubated with one of the primary antibody solutions—anti-fibrinogen (ab92572, 1:200; Abcam), anti-CTGF (ab6992, 1:200; Abcam), or anti-vWF (ab6994, 1:200; Abcam)—overnight at 4 °C, rinsed thoroughly with PBS three times to remove unbound primary antibody, and then incubated with a secondary antibody of donkey anti-rabbit IgG H&L (Alexa Fluor® 594) (ab150076, 1:500; Abcam) for 1 h at 37 °C. Nuclei were stained with Hoechst 33342 (1:1000; Sigma) for 5 min. The sections were disposed with Enhanced Antifade Mounting Medium (Leagene, China) and observed by laser scanning confocal microscopy (Nikon A1R, Japan).

### Sirius red staining

The other frozen bone sections from each group were rewarmed and stained with sirius red (Solarbio, China) according to the manufacturer’s protocol. Type I and type III collagen deposition were evaluated using a polarizing microscope imaging system (Leica, Germany). Type I collagen was colored orange or red and type III collagen was green under the polariscope.

### Statistical analysis

Statistical analysis was performed using SPSS 22.0 software. Data were expressed as the mean ± standard error of the mean (s.e.m.). Comparisons between the two groups were performed using independent-sample *t* tests and correlation analyses. All data demonstrated a normal distribution and similar variation between groups. Statistical significance was defined as *P* ≤ 0.05. All figures were generated using GraphPad Prism Software (Version 6; GraphPad Software, Inc., La Jolla, CA, USA).

## Results

### Implantation of a prevascularized TEBG scaffold into a large segmental bone defect in the femur of EGFP^+^ rats

**Fig. 1:**
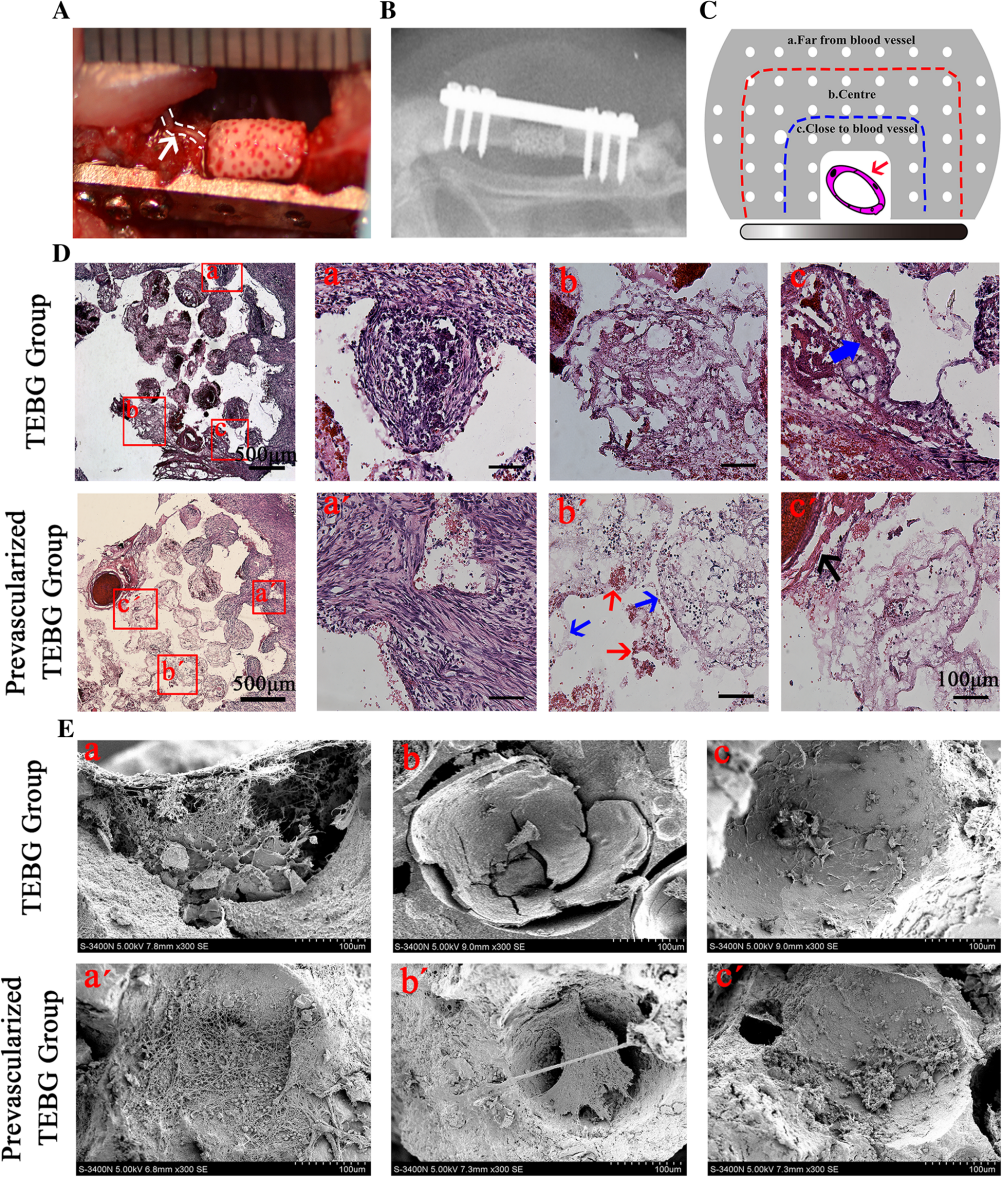
Prevascularization promoted formation of fibrin reticular structure in center of β-TCP and increased cell infiltration. (**A**) Large bone defect model in rat femur. White arrow and curve, implanted vascular bundles (*n* = 6). (**B**) After operation, all rats examined using X-ray imaging to confirm model was successful. (**C**) Schematic diagram of different positions in β-TCP (red arrow, implanted blood vessel): far from blood vessel (a), center of β-TCP scaffold (b), and close to blood vessel (c).(**D**) At 1 week after operation, H&E staining of TEBG sections (a–c) and prevascularized TEBG sections (a′–c′); scale bars = 100 μm. Blue arrows, tubular joints between micropores; red arrows, blood cells attached to reticulate structure; black arrow, implanted blood vessel. (**E**) Representative images under scanning electron microscope (300×) of TEBG group (a–c) and prevascularized TEBG group (a′–c′). TEBG tissue-engineered bone graft

A 5-mm defect of rat femur was regarded as a critical bone defect according to the previous study [22], which could not be repaired by itself. In our study, a 5-mm critical-size bone defect model was established on the femoral shaft of EGFP^+^ rats. In the prevascularized TEBG group, the femoral vascular bundle was inserted into the lateral groove of the scaffold (Fig. 1A). The TEBG group was only implanted into β-TCP as a control.

After operation, X-ray imaging confirmed the steel plates and screws were tightly fixed to the broken end of the bone defect, and the scaffolds did not exhibit slip, indicating the model was established successfully (Fig. 1B). Due to the tight fixation of steel plates, the injured limb of the experimental animal can bear the weight directly after awakening, without affecting their activities, diet, rest, and so on. In addition, the tissue ischemia caused by vascular injury could be compensated by collateral circulation, and hematoma and mild inflammation would last for 1–2 weeks after operation.

TEBGs were obtained at 1 and 4 weeks post operation, and tissue and cell infiltration were assessed in three different locations within the scaffold: far from the blood vessel (> 1200 μm; Fig. 1C, a), β-TCP center (600–1200 μm; Fig. 1C, b), and close to the blood vessel (< 600 μm; Fig. 1C, c).

### Prevascularization promoted the formation of fibrin-like reticular structure in the center of β-TCP and increased cell infiltration

H&E staining and light microscopy were performed to assess the distribution of tissue and cells within the implants at 1 week post transplantation. Insertion of the vascular bundles into the scaffolds could be clearly observed in the prevascularized TEBG group.

The micropores of the prevascularization scaffolds were filled with reticular structure. In contrast, there were large blank areas in the center of the TEBG group (Fig. 1D). There were significant differences in cell status and extracellular matrix deposition at different locations of the two groups. A large number of inflammatory cells were located far from the vascular bundle in the TEBG control group, but the cells of the prevascularization TEBG group were arranged regularly and well in shape (Fig. 1D, a and a′). There were many fibrin-like reticulation and tubular connections between pores in the β-TCP center of the prevascularization group; simultaneously, a large number of blood cells attached to the network structure and the connecting tube, and these blood cells may provide oxygen for exogenous seed cells to increase their survival rate. On the contrary, there were some blank areas within the control sections, indicating that the extracellular matrix and cells had not yet infiltrated into the material center (Fig. 1D, b and b′). Reticular structures were also distributed around the vascular bundle in the prevascularized scaffolds. In contrast, cord-like structures created by the extension of the surrounding callus were observed around the edges of the control scaffolds (Fig. 1D, c and c′), but these cord-like structures only existed around the callus and did not extend to the center of the scaffolds. The results from the scanning electron microscope were consistent with the histopathological analysis mentioned earlier. There was almost no cell infiltration into the center of the TEBG group so that materials dry and crack (Fig. 1E, a–c). However, scanning electron microscopy (SEM) confirmed the reticulated, fibrin-like structure and that numerous blood clots were present throughout all three zones of the prevascularized scaffolds (Fig. 1E, a′–c′).

Surgical trauma causes blood vessel rupture, bleeding, and hematoma formation, and hematoma gradually transformed into a blood clot. New blood capillaries, fibroblasts, and phagocytic cells invaded the blood clot within 24 h. At the same time, platelets, disintegrating tissue, and perivascular cells released some cytokines involved in bone repair, such as platelet-derived growth factor (PDGF), transforming growth factor (TGF-β), and so on [23, 24]. Clots would not always exist and are gradually cleared in later stages and quickly became granulation tissue, which in turn formed fibrous callus [25]. Therefore, in the early stage of bone defect, there would be some blood clots blocking the scaffold’s pores. However, with the destruction of erythrocytes in the hematoma, fibrin exudes and blood clots were replaced by a fibrin network structure. Thus, our data indicated that prevascularization promoted formation of a fibrin network structure within the scaffolds, which provided a support and bridge for endogenous cell migration between the micropores of the material.

### Prevascularization increased the expression of fibrinogen, and then promoted EGFP^+^ endogenous cell infiltration

**Fig. 2:**
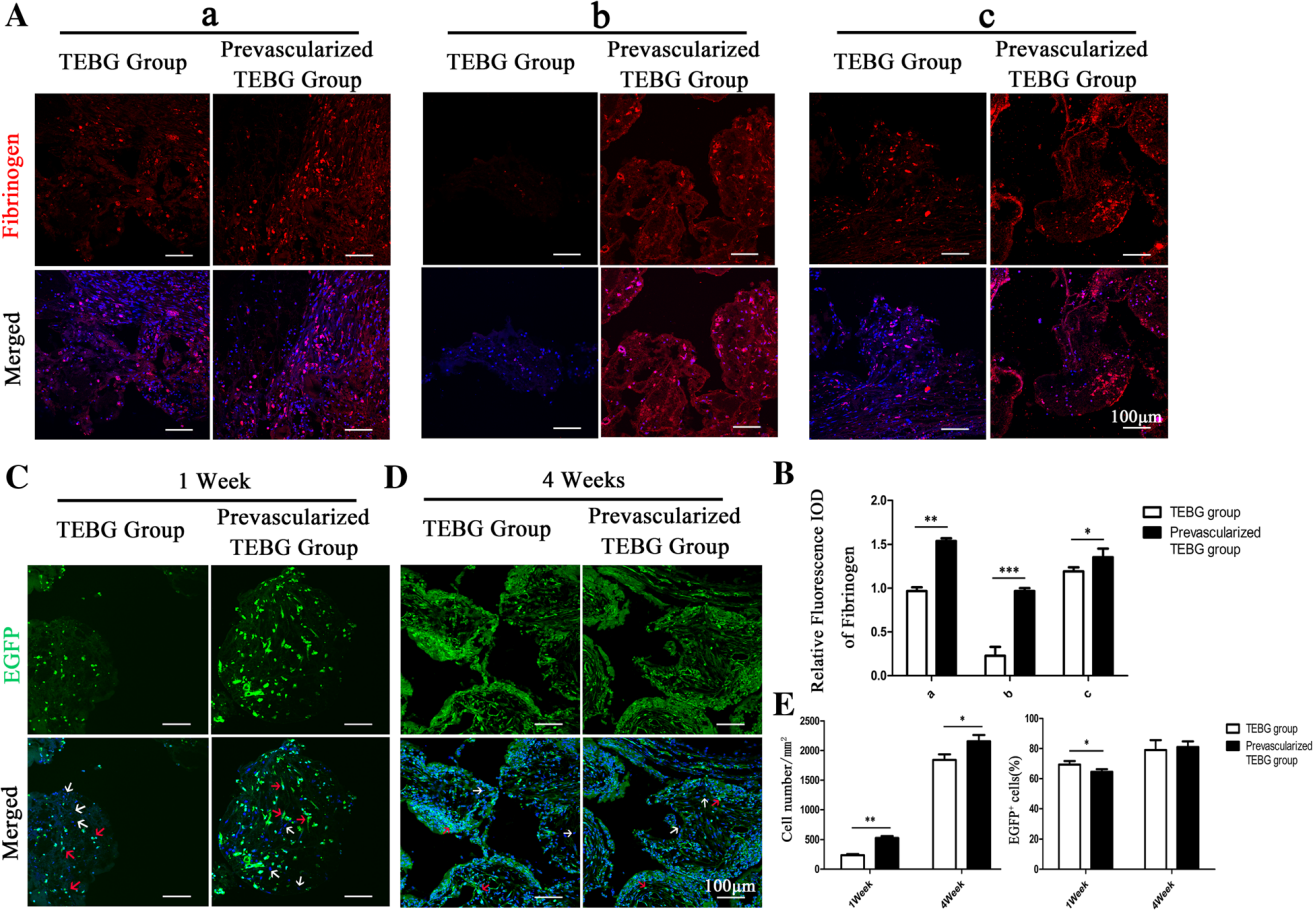
Prevascularization increased expression of fibrinogen, and then promoted EGFP^+^ endogenous cell infiltration. (**A**) At 1 week post operation, immunofluorescence images of fibrinogen (red) and Hoechst 33342 (blue) from TEBG sections and prevascularized TEBG sections: far from blood vessel (a), β-TCP center (b), and close to blood vessel (c); scale bars = 100 μm. (**B**) Relative fluorescence integrated optical density (IOD) of fibrinogen, *n* = 5. (**C, D**) Immunofluorescence images of endogenous cells (EGFP^+^, green) and Hoechst 33342 (blue) in β-TCP center at 1 week (**C**) and 4 weeks (**D**) after operation. Red arrows, endogenous cells of EGFP^+^; white arrows, exogenous cells of EGFP^−^; scale bars = 100 μm. (**E**) Number of total cells per mm^2^ and percentage of EGFP^+^ cells. *n* = 5. Data presented as mean ± s.e.m. *P < 0.05, **P < 0.01, *** *P* < 0.001 determined by Student’s *t* test. EGFP enhanced green fluorescent protein, TEBG tissue-engineered bone graft

In order to confirm whether insertion of the vascular bundle promoted fibrin production, we examined the expression of fibrinogen by immunofluorescence staining (Fig. 2A, B). The relative fluorescence integrated optical density (IOD) of fibrinogen in the prevascularization TEBG group was significantly higher than in the TEBG group in all three zones of the scaffold: close to and far from the vascular bundle, and especially in the center of β-TCP. Thus, the immunofluorescence staining confirmed the histopathology and SEM observations that prevascularization promoted formation of a reticular fibrin network within the materials.

In this model, it was possible to distinguish between endogenous cells (EGFP^+^) and exogenous cells (EGFP^−^) by tracing the green fluorescent protein markers. Thus, we explored whether this reticular structure promoted infiltration of endogenous cells and survival of exogenous seed cells. At 1 and 4 weeks post operation, prevascularization can significantly increase the total number of cells in the materials (Fig. 2C–E). At 1 week, the number of endogenous cells in the prevascularized group was more than twofold higher than the number of cells in the TEBG group. However, the proportion of EGFP^+^ endogenous cells in the total number of cells was lower than that of the control group, indicating that vascular bundle implantation significantly promoted the survival of exogenous seed cells (Fig. 2E).

Collectively, these analyses indicated that the fibrin network within the prevascularized scaffolds provided a structural connection between the internal micropores of the scaffold, which supported endogenous cell infiltration and migration, which may facilitate formation of a vascular network to supply nutrients and oxygen to the exogenous seed cells and increase their survival rate.

### Prevascularization increased the expression of CTGF

**Fig. 3:**
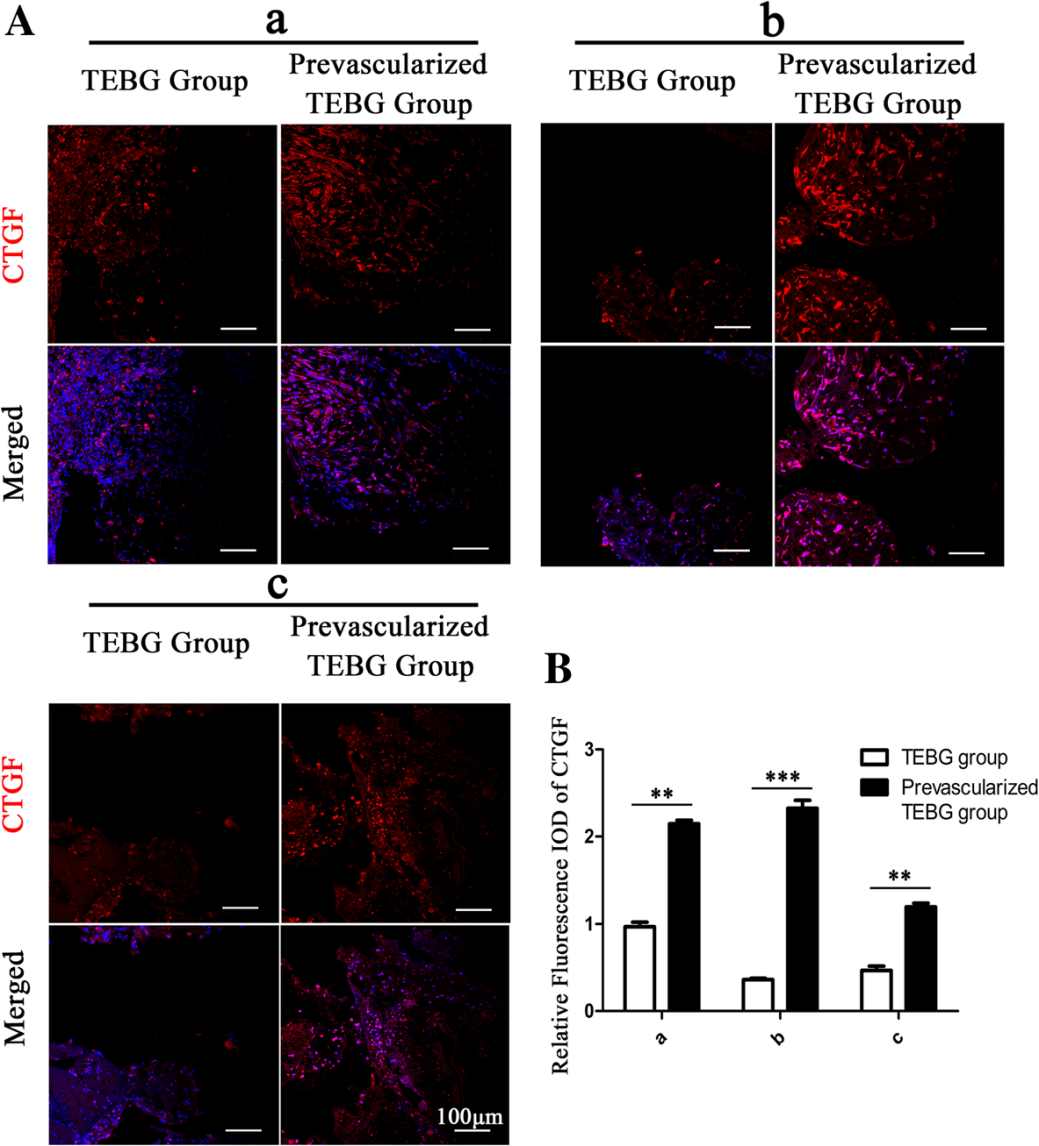
Prevascularization increased expression of CTGF. (**A**) At 1 week post operation, immunofluorescence images of CTGF (red) and Hoechst 33342 (blue) from TEBG sections and prevascularized TEBG sections: far from blood vessel (a), center (b), and close to blood vessel (c); scale bars = 100 μm. (**B**) Relative fluorescence integrated optical density (IOD) of CTGF, *n* = 5. Data presented as mean ± s.e.m. **P < 0.01, *** *P* < 0.001 determined by Student *t* test. CTGF connective tissue growth factor, TEBG tissue-engineered bone graft

CTGF is a modular secreted protein closely associated with multiple cellular events such as chondrogenesis, skeletogenesis, trauma repair, and angiogenesis [26]. Under physiological conditions, CTGF appears to have a role in collagen synthesis, and to accelerate the production of extracellular matrix and support the newly formed vascular structure to promote angiogenesis [27]. Therefore, we assessed the expression of CTGF at 1 week after implantation by immunofluorescent analysis of frozen sections. The results showed that prevascularization significantly increased the distribution areas and relative IOD of CTGF in all three zones of the grafts (Fig. 3A, B). A high level of CTGF expression is likely to facilitate recruitment of cells, and thus CTGF may promote infiltration of endogenous cells into tissue-engineered bone grafts, to promote angiogenesis and accelerate bone repair.

### Prevascularization enhanced the deposition of collagen type I/III within the scaffold

Collagen type I is the main component of bone tissue, contributing to the elasticity and toughness of bone. Collagen type III is a reconstituted collagen that plays an important role in tissue damage repair. It can directly promote angiogenesis and maintain the function of capillaries.

**Fig. 4:**
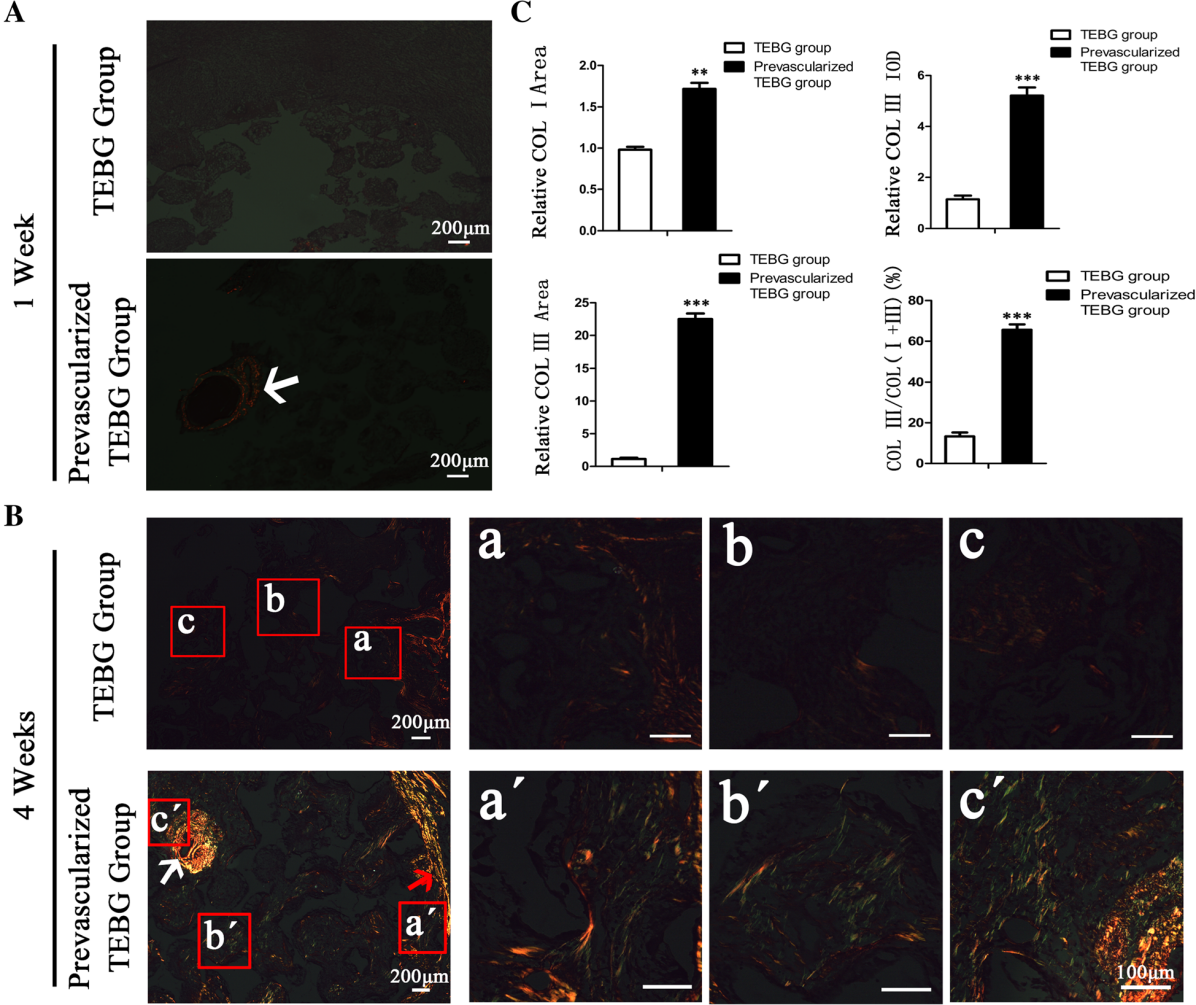
Prevascularization promotes synthesis of collagen type I/III. (**A**) At 1 week after operation, sirius red staining in tissue section. White arrow, implanted blood vessel; scale bars = 200 μm. (**B**) Sirius red staining of TEBG sections (a–c) and prevascularized TEBG sections (a′–c′) at 4 weeks; scale bars =100 μm. White arrow, implanted blood vessel; red arrow, region near callus. (**C**) Relative deposition area of type I and type III collagen, relative IOD of type III collagen, and ratio of type III collagen to the total area of type I and type III collagen, *n* = 4. Data presented as mean ± s.e.m. **P < 0.01, *** *P* < 0.001 determined by Student *t* tests. COL collagen, TEBG tissue-engineered bone graft

Our results indicated that vascular bundle implantation increased endogenous cell infiltration and exogenous cell survival. Thus, we explored whether these cells within the scaffold secrete matrix and participated in tissue reconstruction. Sirius red staining showed no obvious collagen deposition within the two groups at 1 week after implantation. Interestingly, a high level of type I collagen expression was observed around the implanted vascular bundle in the prevascularized TEBG group (Fig. 4A).

At 4 weeks after the operation, prevascularized scaffolds contained higher levels of both collagen type I and type III close to the vascular bundle, far from the vascular bundle, and in the center of the scaffold compared to control scaffolds (Fig. 4B, C). In addition, the proportion of type III collagen relative to total collagen was significantly higher in prevascularized TEBG scaffolds than in TEBG scaffolds.

Dynamic interactions between extracellular matrix (ECM) and growth factors are integral to wound healing. These interactions take several forms that may be categorized as direct or indirect. Collagen III is one of the ECM components that can directly bind to or release certain growth factors. Additionally, the collagen can bind to cell surface receptors in the cytokine, chemokine, or growth factor families and stimulate cellular activities [28]. Collagen III has been widely used to facilitate cell growth and differentiation in constructive remodeling [29]. In summary, collagen III interacts with various cells and growth factors, which enhances communication and interaction between cells and accelerates bone defect repair.

### Prevascularization promoted formation of vWF^+^ capillary-like tubular connections between the scaffold micropores

**Fig. 5:**
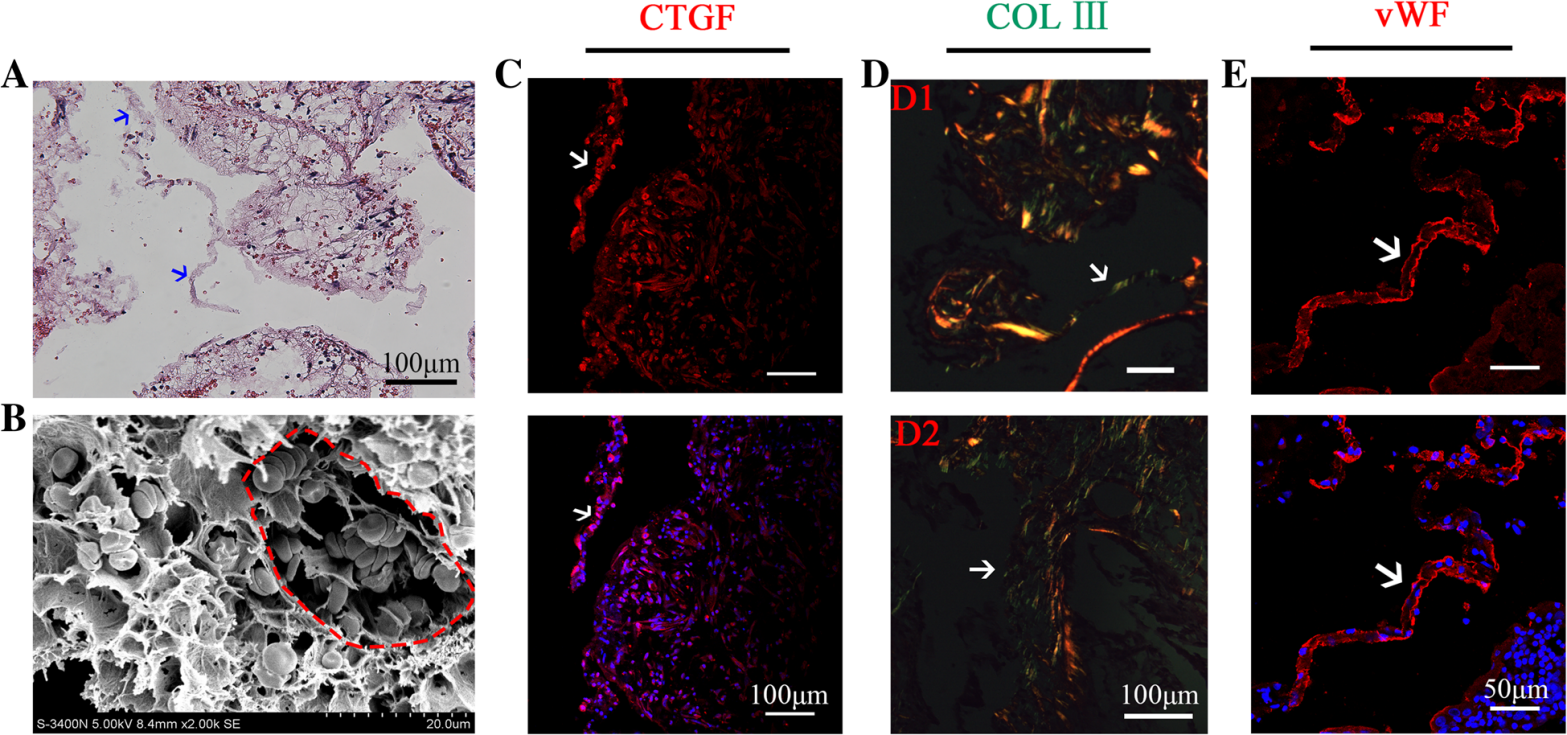
Fibrin constituted tubular connection between pores, which expressed CTGF in early stage, and later transformed into capillary-like structure of vWF^+^ with type III collagen deposition. **a** H&E staining. Blue arrow, tubular structure of fibrin between micropores; scale bars = 100 μm. **b** SEM images (2000×) show tubular structure provided a scaffold for cell attachment. Red dotted circle, tubular structure. **c** Immunofluorescence images of CTGF (red) at 1 week post operation. White arrows, expression of CTGF in tubular structure; scale bars = 100 μm. **d** Sirius red staining at 4 weeks. White arrows, type III collagen deposited in tubular structure at early (D1) and later (D2) stages of connection; scale bars =100 μm. **e** Immunofluorescence images of CTGF (red) at 4 weeks. White arrow, capillary-like structure of vWF^+^; scale bars = 50 μm. COL collagen, CTGF connective tissue growth factor, vWF von Willebrand factor

The connection between micropores in porous materials plays a crucial role in tissue repair and material degradation, such as supporting cell adhesion, migration, and interaction, and promoting matrix deposition. H&E staining suggested that the reticulated fibrin structure promoted cell migration and construction of tubular connections between the pores within β-TCP (Figs. 1D and 5a). SEM revealed that numerous blood cells were attached to the tubular structure (Fig. 5b), in accordance with the histological staining (Fig. 1D, b′). Further study found high expression of CTGF in the tubular area at 1 week after surgery (Fig. 5c), which promoted the recruitment of increased numbers of endogenous cells to this region, facilitating cellular communication and delivery of nutrients between the micropores. Furthermore, the recruited cells secreted type III collagen that filled the tubular structure (Fig. 5d), and eventually transformed into vWF^+^ capillary-like structure at 4 weeks after implantation (Fig. 5e). In addition, the diameter of these vWF^+^ structures was 5–10 μm, which coincided with the diameter of capillaries [30–32].

These results suggested that the tubular structure of fibrin, which is gradually transformed into a vascular structure due to the action of factors such as CTGF and type III collagen, and the tubular fibrin network provided a bridge to the connect the micropores and promote vascularization of β-TCP.

### Prevascularization promoted the angiogenesis of endogenous cells

**Fig. 6:**
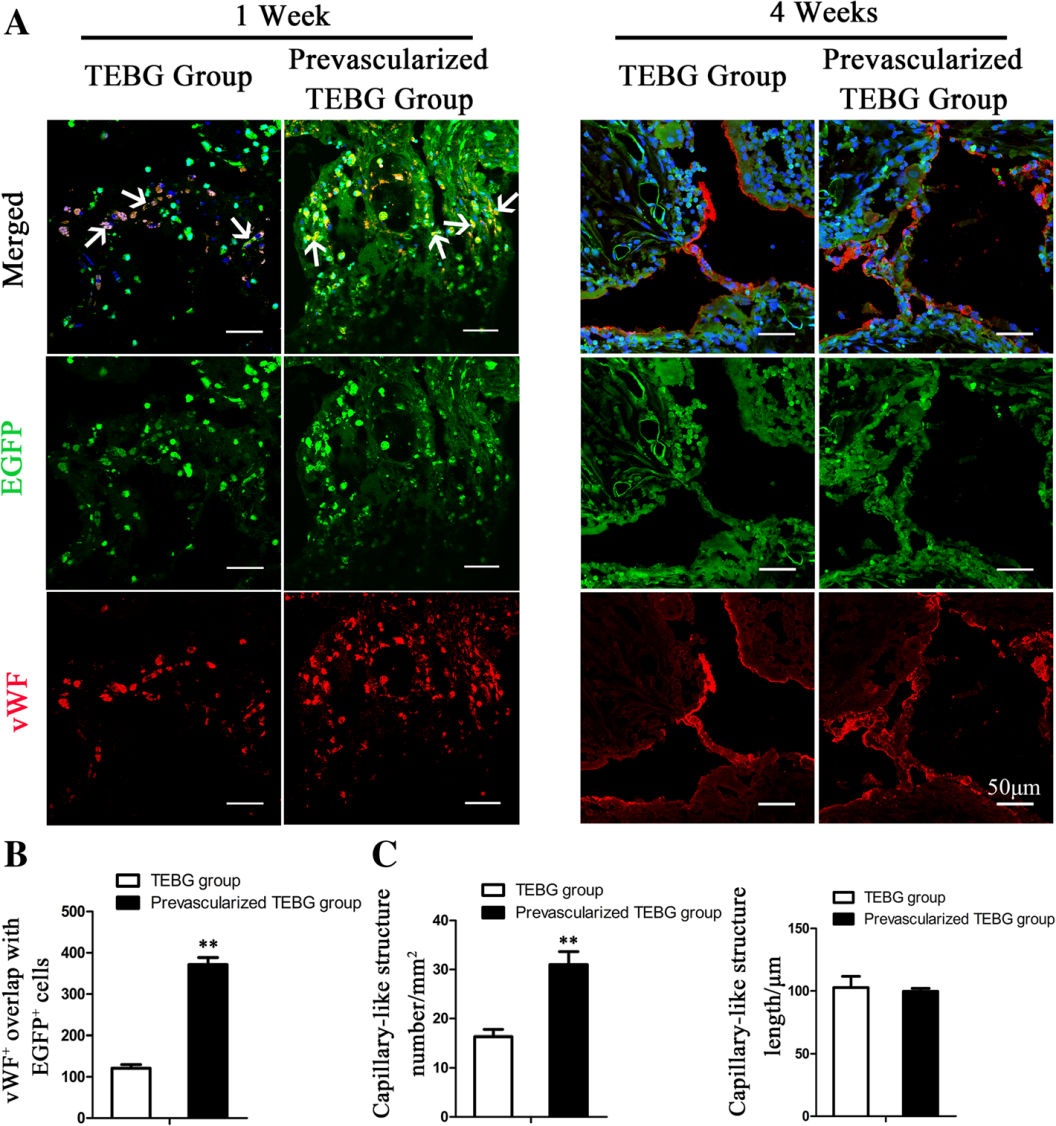
Prevascularization increased number of vWF^+^ endogenous cells and neonatal capillary-like structures. **a** Immunofluorescence images of vWF (red) and EGFP (green) from TEBG sections and prevascularized TEBG sections at 1 and 4 weeks after transplantation. White arrows, double-positive staining cells. Hoechst 33342 stained nuclei blue; scale bars = 50 μm. **b** Number of vWF^+^ cells overlaid with EGFP^+^ endogenous cells, *n* = 6. **c** Number of capillary-like structures (mm^2^) and length (μm), *n* = 6. Data presented as mean ± s.e.m. **P < 0.01 determined by Student *t* tests. EGFP enhanced green fluorescent protein, TEBG tissue-engineered bone graft vWF von Willebrand factor

Endogenous cells play a more important role in tissue damage repair than exogenous cells. We explored the effect of prevascularization on endogenous cell-mediated angiogenesis by fluorescence colocalization of EGFP and vWF. At 1 week post operation, prevascularization can significantly increase the number of endogenous cells in vWF^+^ (Fig. 6a, b), which may be involved in tissue reconstruction and angiogenesis. At 4 weeks after surgery, vWF^+^ tubular structures were present between the micropores of the material. The number of these capillary-like structures in the prevascularization group was significantly higher than in the TEBG group. However, there was no obvious difference in capillary length (Fig. 6a, c), which may be related to the relatively uniform distance between the micropores of the material.

### Prevascularization promoted bone regeneration

At 4 weeks after surgery, prevascularization significantly promoted tissue ingrowth and material degradation. At 8 weeks, there was obvious new bone formation at the edge of material pores (Additional file 1: Figure S1A). H&E staining, X-ray, and micro-CT results showed that vascular bundle implantation significantly accelerated the process of bone repair (Additional file 1: Figure S1B, C). In addition, immunofluorescence staining showed that the number of osteoblasts (Osterix^+^) in the prevascularization group was significantly higher than in the TEBG control group (Additional file 1: Figure S1D, E). It is interesting that both endogenous and exogenous cells (BMSCs) are involved in the process of bone regeneration (Additional file 1: Figure S1D).

**Fig. 7:**
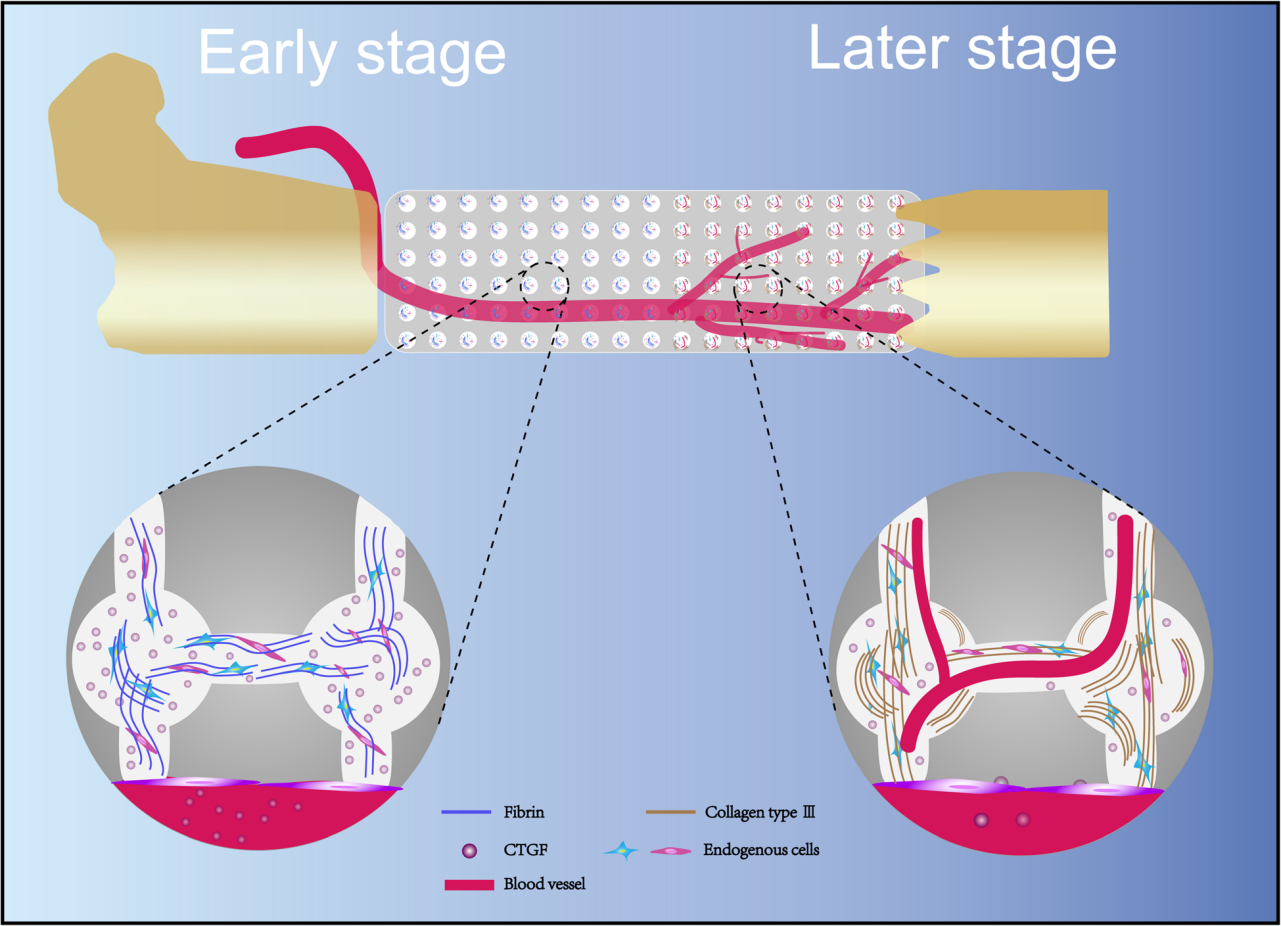
Prevascularization promoted endogenous cell-mediated angiogenesis in TEBG. Fibrin content increased at early stage of vascular implantation, and reticular formation of fibrin provided scaffold for endogenous cell infiltration and created a tubular connection between pores. In addition, prevascularization improved expression of CTGF, and then promoted collagen synthesis and angiogenesis (left panel). In the later stage, these tubular connections transformed into vWF^+^ capillary-like structures with type III collagen deposition, eventually increasing number of new blood vessels (right panel). CTGF connective tissue growth factor

In summary, the results of this study showed that in the early stage of vascular bundle implantation, the content of fibrinogen increased, forming a fibrin reticular structure and a connecting tube between micropores, providing scaffolds for cell attachment and migration. Meanwhile, CTGF expression is increased, and more endogenous cells such as vascular endothelial cells can be recruited to participate in bone defect repair (Fig. 7, left panel). In the later stage, these tubular connections transformed into vWF^+^ capillary-like structures with type III collagen deposition, and promoted neovascularization by endogenous cells (Fig. 7, right panel). These nascent capillaries provided nutritional support to maintain cell survival and function, thereby accelerating the process of bone defect repair.

## Discussion

We reported previously that TEBGs prevascularized by inserting a vascular bundle significantly increased the number of capillary vessels. In this study, we aimed to identify the mechanism by which prevascularization promotes vascularization of the TEBG.

Since the 1990s, tissue engineering has undergone many advances in scaffold development, seeding cells, animal models, and clinical trials [33, 34]. Some previous studies have shown that endogenous cells are the main contributors to tissue reconstruction and angiogenesis; they play a more important role than exogenous cells in the process of defect repair [17, 18, 35]. However, most studies have focused on the survival and function of exogenous seed cells, with little attention given to the distribution and behavior of endogenous cells. Our study successfully generated a 5-mm-length defect in rat femurs using customized internal fixation plates. With the use of EGFP^+^ transgenic rats, this large defect model provided the possibility to further explore the contribution of endogenous cells and exact molecular mechanisms by tracing EGFP.

Our observations indicated that insertion of the vascular bundle into the scaffold before implantation led to accumulation of fibrinogen within the TEBG. Fibrinogen, a major plasma protein that plays a key role in hemostasis, is converted to fibrin in response to bone injury [36, 37], and the resulting reticulated fibrin network created cross-linked, tube-like connections between the micropores of the scaffold at 1 week after implantation. This fibrin network appeared to provide a substrate for cell adhesion to facilitate exogenous seed cell survival and endogenous cell migration into the scaffold. Histology, SEM, and EGFP fluorescence tracing confirmed that endogenous cells infiltrated into the TEBGs immediately after implantation. Moreover, the fibrin network promoted exogenous seed cell survival and also significantly increased the number of endogenous cells that migrated and/or proliferated within the scaffold. (Fig. 2C–E). Similarly, recent articles have reported that fibrinogen scaffolds significantly promote vascularization in vivo [38, 39].

Fibrin polymers are widely used in the tissue engineering field as biomaterials. The fibrinogen content of fibrin glue/bone powder scaffolds would be benefit bone tissue engineering [40]. Combining platelet-rich fibrin (PRF) with the synthetic material (the combination of hydroxyapatite and β-TCP) resulted in more cortical and subcortical bone formation [41]. Fibrin preparations can support the proliferation and growth of periosteal cells to form well-combined active biological materials [42]. Therefore, fibrin could be clinically applicable in bone regeneration therapy.

In addition, prevascularization induced high-level CTGF expression within the scaffolds at 1 week after implantation. CTGF (also known as CCN2), a cysteine-rich protein that is widely expressed in response to tissue damage, can be synthesized by fibroblasts, smooth muscle cells, and endothelial cells. CTGF has been shown to promote cell adhesion, migration, and proliferation [26]. CTGF exerts proangiogenic activity and interacts with other proangiogenic proteins via several mechanisms to regulate angiogenesis [27], and has also been reported to promote tumor angiogenesis [43, 44] Liu et al. [45] suggested that CTGF significantly increased angiogenesis regulated by vascular endothelial growth factor (VEGF) in human synovial fibroblasts. Grote et al. [46] showed that CTGF recruited CD34^+^ progenitor cells and thereby promotes endothelial cell proliferation and capillary formation.

CTGF also promoted collagen synthesis [47–49], which may explain the increased deposition of type I and type III collagen in the prevascularized scaffolds at 4 weeks post operation (Fig. 4B). The components of the ECM, such as collagen type I and III, play vital roles in the formation of new blood vessels [50, 51]. Type I collagen is the main component of bone tissue, and type III collagen is expressed at high levels during remodeling of injured tissue [50, 52]. Type III collagen is also abundant in blood vessels, where it contributes to the strength and elasticity of capillaries.

Fibrin and collagen differentially, but synergistically, regulate angiogenesis [7]. The fibrin network that formed between the pores of the scaffold expressed CTGF at 1 week after implantation, and had transformed into vWF^+^ capillary-like structures with deposition of type III collagen by 4 weeks after implantation (Fig. 5), which in turn promoted neovascularization by endogenous cells (Fig. 7, right panel). Fluorescence microscopy confirmed that prevascularization promoted angiogenesis mediated by endogenous cells within the TEBGs. These nascent capillaries are likely to provide nutritional support to maintain cell survival and function, and thereby accelerate bone defect repair processes. Overall, this study suggests prevascularization induces angiogenesis within the scaffold via a mechanism involving fibrinogen/fibrin–CTGF–type III collagen, as illustrated in Fig. 7.

The exogenous cells used in this experiment were purified BMSCs (CD90^+^, CD34^−^, CD45^−^, CD11b/c^−^), which could differentiate into osteoblasts, cartilage, and adipocytes in vitro [21, 34]. BMSCs have low immunogenicity and can induce a transient immunoreaction in early stage after transplantation, whereas the long-term engineered bone formation was not affected [53, 54]. Moreover, BMSCs induce T-cell hyporesponsiveness and prolong graft survival in the rat vascularized composite allotransplantation model. BMSCs exhibit immunomodulatory properties against acute rejection that can be realized without the need for significant recipient immunosuppression [55].

Correspondingly, no obvious rejection was found in our experimental animals, and only mild inflammation would last for 1–2 weeks after surgery, which may be caused by surgical trauma. In addition, inflammation in the prevascularization group was lower than in the TEBG group.

One limitation of this study is that all endogenous cells expressed EGFP. Therefore, the key role of specific cells is unknown. Further in-vitro and in-vivo experiments should be designed to further explore the precise roles and molecular mechanisms of action of individual types of endogenous. Furthermore, we divided the grafts into three regions: far from the vascular bundle, close to the vascular bundle, and the material center. The results showed that the histopathological morphology, inflammation levels, and repair process varied at different locations, but the mechanism for such a significant difference still needs further study.

In summary, this exploration of the effect of prevascularization on endogenous cells provided a new perspective for the study of TEBG vascularization, which may also help to develop more molecular-based drugs and materials, thus replacing implantation of the vascular bundle and increasing the efficiency of neovascularization within TEBGs.

## Conclusions

The contribution of endogenous cells in tissue reconstruction and functional regeneration of bone injury is greater than that of exogenous seed cells. How to improve the infiltration speed and quantity of endogenous cells is important for bone and blood vessel regeneration. In this paper, EGFP transgenic rats were used to establish a model of large femoral bone defect. For the first time, we found that the implantation of vascular bundles could increase the expression of fibrinogen and CTGF, and then the fibrin reticular scaffold formed by fibrinogen significantly increased the number of vascular endothelial cells and other endogenous cells. Finally, with the deposition of type III collagen, a more capillary-like structure of vWF^+^ was formed within the material, thus promoting vascularization of the TEBGs. In addition, this molecular mechanism can be used to establish fast-acting angiogenesis materials in future clinical applications.

## Additional file

Additional file 1: **Figure S1.** Prevascularization promoted bone regeneration in TEBG. (**A**) H&E staining of TEBG sections and prevascularized TEBG sections at 4 weeks and 8 weeks post operation. Black arrow, implanted blood vessel. Red arrows, regenerative bone tissue. (**B**) At 8 weeks, X-ray imaging analysis of bone defect repair and micro-CT 3D reconstruction images of new bone formation. (**C**) BV/TV used to evaluate new bone formation. *P < 0.01 determined by Student *t* tests. (**D**) Immunofluorescence images of Osterix (red) and EGFP (green) from TEBG sections and prevascularized TEBG sections at 8 weeks after transplantation. Hoechst 33342 stained nuclei blue; scale bars = 50 μm. Red arrows, endogenous osteoblasts; white arrows, exogenous-derived osteoblasts; scale bars = 20 μm. (**E**) Total number of Osterix^+^ cells. **P < 0.01 determined by Student *t* tests. (TIF 9451 kb)
