# Supplementary figures and images for: Prevascularization promotes endogenous cell-mediated angiogenesis by upregulating the expression of fibrinogen and connective tissue growth factor in tissue-engineered bone grafts
Source: Stem Cell Res Ther. 2018 Jul 4;9:176. doi: 10.1186/s13287-018-0925-y (PMC6030739; doi:10.1186/s13287-018-0925-y)

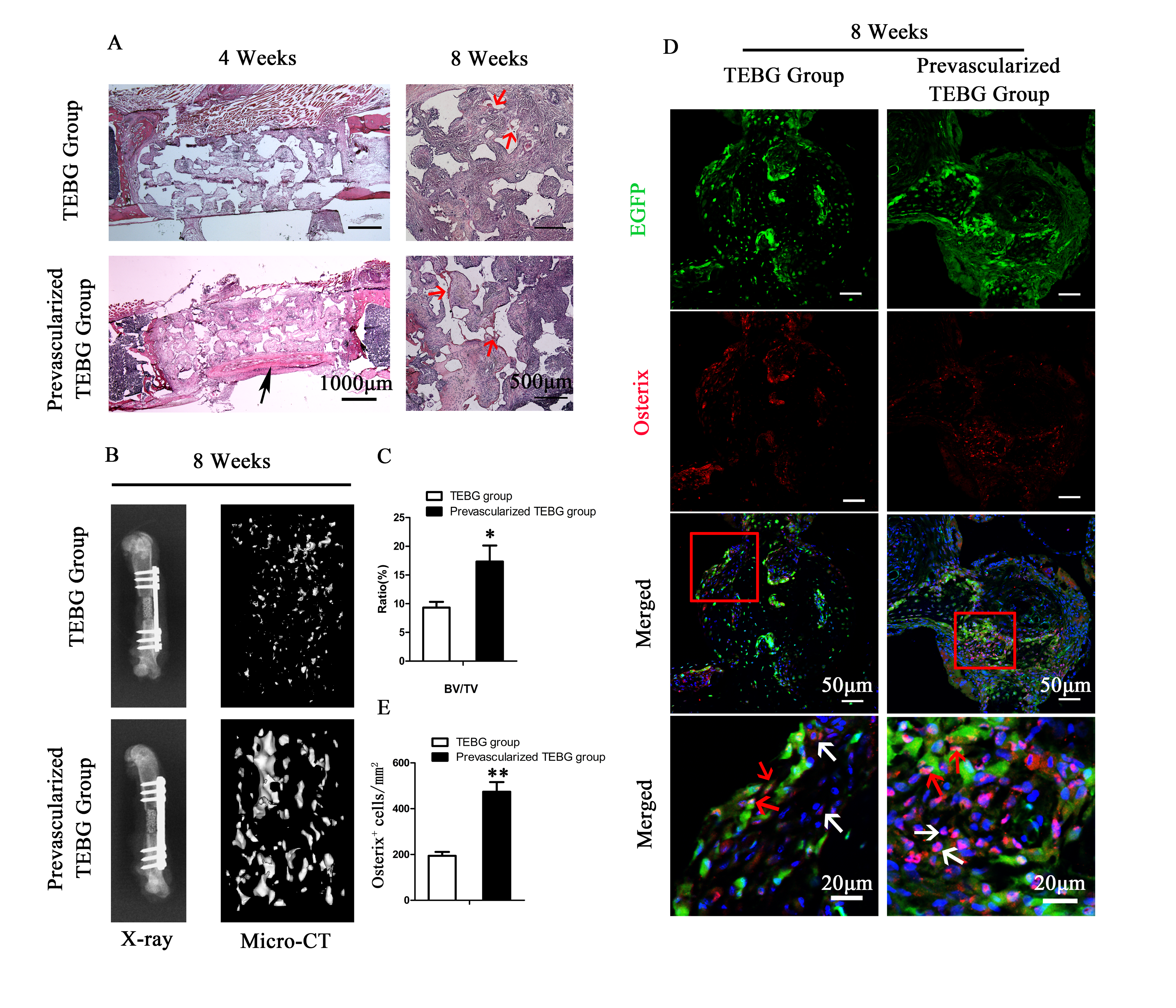

Supplement: Supplementary file 1 — Figure S1. Prevascularization promoted bone regeneration in TEBG. (A) H&E staining of TEBG sections and prevascularized TEBG sections at 4 weeks and 8 weeks post operation. Black arrow, implanted blood vessel. Red arrows, regenerative bone tissue. (B) At 8 weeks, X-ray imaging analysis of bone defect repair and micro-CT 3D reconstruction images of new bone formation. (C) BV/TV used to evaluate new bone formation. *P < 0.01 determined by Student t tests. (D) Immunofluorescence images of Osterix (red) and EGFP (green) from TEBG sections and prevascularized TEBG sections at 8 weeks after transplantation. Hoechst 33342 stained nuclei blue; scale bars = 50 μm. Red arrows, endogenous osteoblasts; white arrows, exogenous-derived osteoblasts; scale bars = 20 μm. (E) Total number of Osterix+ cells. **P < 0.01 determined by Student t tests. (TIF 9451 kb) [file 13287_2018_925_MOESM1_ESM.tif]
